# The future of non‐financial businesses reporting: Learning from the Covid‐19 pandemic

**DOI:** 10.1002/csr.2145

**Published:** 2021-05-20

**Authors:** Abeer Hassan, Ahmed A. Elamer, Suman Lodh, Lee Roberts, Monomita Nandy

**Affiliations:** ^1^ Accounting Finance and Law division, School of business and creative industry University of the West of Scotland Paisley UK; ^2^ Brunel Business School Brunel University London Uxbridge UK; ^3^ Accounting and Finance Department, The School of Business and Law Middlesex University London UK

**Keywords:** biodiversity, business reporting, circular economy, integrated reporting, Covid‐19 crisis, non‐financial reporting, stakeholder engagement, sustainable development

## Abstract

In this paper we conceptually identify the gap in the literature about lack of business's awareness in non ‐financial activities, especially biodiversity, which can be responsible for crisis like Covid‐19 which can adversely affect the global economy. We recommend approaches to existing business about how to enhance the quality of reporting by considering non‐human element in reporting and making it more comprehensive for the stakeholders. We adopt Actor Network Theory (ANT) and the Natural Inventory Model to support our argument that nature consists of both human and non‐human. From our observation about the Covid‐19 crisis and by consulting the existing relevant literature on CSR, Covid‐19, non‐financial reporting and integrated reports (IR), we propose the implication of non‐financial reporting by companies based on a theoretical framework. We recommend that companies should implement/adopt Circular Economy concept for sustainable business model and report on biodiversity and extinction accounting in more structured and mandatory way via producing IR to create value on short, medium and long terms. This is the first paper to tackle the Covid‐19 crisis and offer solution for future reporting. The findings will add value in the academia and society.

## 1 INTRODUCTION

**FIGURE 1:**
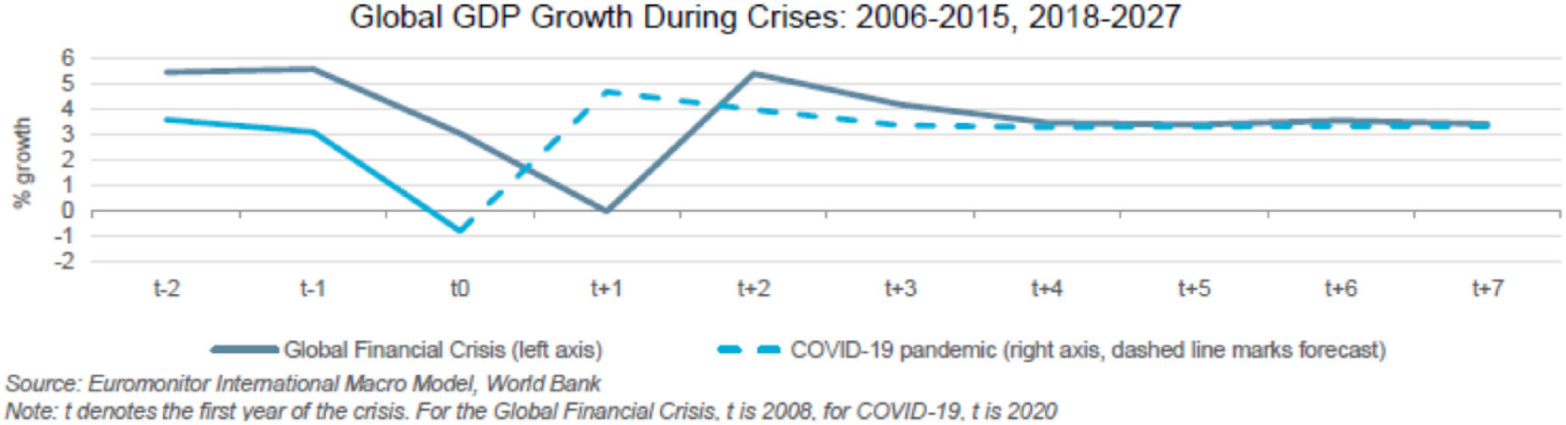
*Source:* Passport: Economic Outlook [Colour figure can be viewed at wileyonlinelibrary.com]

The United Nations (UN) mention that there is an immense need to develop a resilient and sustainable all‐inclusive global system in the post Covid‐19 period (UN.org, 2020). In response to this call from the UN, this research note highlights how the company's accountability towards society can positively affect the economy to face future global challenges, like the Covid‐19 pandemic. The impact of coronavirus disease in 2019 (Covid‐19)^1^ on society is shocking and the global economy is affected quicker and more severely than the 2007–2009 global financial crisis and the Great Depression (Roubini, 2020)^2^ for a number of reasons (see Figure 1). First, at the time of the composing of this research note, the unprecedented Covid‐19 epidemic has already affected over 119,904,724 people and killed over 2,655,650 people worldwide (World Bank, 2020). Second, Covid‐19 has affected all factors of aggregate demand (i.e., consumption, capital spending, and exports) in exceptional decline. Third, policymakers in less than a month put in place a massive fiscal stimulus to businesses and households compared to the reactions that took around 3 years when the global financial crisis happened. However, during the Covid‐19 crisis, we find that many companies have extensively engaged in social responsibility (hereafter CSR) and consider society as a stakeholder in their business model, even when resources were scarce (Fairshare.org.uk, 2020).

It is evident in the literature that when a company cares towards society it creates a long‐lasting social identity which generates brand loyalty associated with long term profits (García‐Sánchez, Hussain, et al., 2019; García‐Sánchez, Martínez‐Ferrero, et al., 2019; García‐Sánchez & Noguera‐Gámez, 2017; He et al., 2012; Hou, 2019; Khatib et al., 2021; Martínez‐Ferrero et al., 2015; McLaughlin et al., 2019; Mio et al., 2020; Muserra et al., 2020; Pistoni et al., 2018; Roberts et al., 2021; Romito & Vurro, 2021). It is observed that these socially accountable businesses managed to survive the Covid‐19 crisis. But on the other hand, we find that many companies are out of business during the Covid‐19 pandemic and these companies were not very active in their social responsibilities. Such contrast in the business world motivated us to examine if the companies considering society as their stakeholder in their business, are they able to manage to survive the adverse economic impact of Covid‐19? The ability to be socially responsible cannot be developed in a short period. Thus, there is a high possibility that the companies that managed to survive the adverse impact of Covid‐19, were presenting their awareness about the environment before the crisis in their company activities. Companies usually disclose their corporate social responsibilities to wider stakeholders through financial reports of the companies. The above argument insisted us to highlight the importance of the reporting of non‐financial aspects like biodiversity etc. by the companies. We want to draw attention of the corporates who are not considering society as a stakeholder in the business and explain to them the importance of non‐financial business reporting as a survival mechanism from any future pandemics. As a result, this research note (1) identifies what changes the business should adopt in their annual reporting (2) Critically discusses the gaps in the extant literature about lack of business's awareness in non‐financial activities, especially biodiversity, which can be responsible for a recent crisis like the one generated by Covid‐19. (3) Recommend how to enhance the quality of reporting and making it more comprehensive for the stakeholders.

In the literature we find that a company can influence the firm value positively by improving disclosure practices as there is a reduction in information asymmetry between agents and managers of the company (Adhikariparajuli et al., 2020; Alnabsha et al., 2018; Hassan et al., 2019; Sheu et al., 2010). The reduced information asymmetry can lead to lower agency cost, which may be the reason behind the company survival during the pandemic. Such disclosure about the responsibility towards society in the business report will require more than International Financial Reporting Standards (IFRS) and climate risk disclosures requirements. However, in the literature, there is a gap about how the disclosure related to the nature in the business report can assist companies to survive any “Black Swan Event” (Taleb, 2008) like Covid‐19 Pandemic? Consistent with the above argument, an emerging stream of the literature suggests that companies must recognise the nature and the ecosystems either directly or indirectly and link them to the corporate activities because of their intrinsic value (Adler et al., 2018; Atkins & Atkins, 2018; Bebbington & Unerman, 2018; Hassan, Roberts, et al., 2020). In addition, to the above, it is worth noting that biodiversity is precarious to businesses' survivals as companies have a two‐way relationship with biodiversity, including both the impact of companies on biodiversity, and the impact of biodiversity on companies (Adler et al., 2018; Hassan, Nandy, & Roberts, 2020). Therefore, companies must recognise that nature and ecosystems are of immense value associated with corporate activities (Adler et al., 2018; Atkins & Atkins, 2018; Bebbington & Unerman, 2018). However, several researchers from different academic disciplines confirm that pandemics are a result of biodiversity loss and habitat destructions (UN, 2020). It is also confirmed that Covid‐19 is the consequence of human persistent and excessive intrusion in nature (World Health Organisation, 2020).

In 2019, The Global Assessment of the Intergovernmental Platform on Biodiversity and Ecosystem Services (IPBES), mentioned how biodiversity is lost in various places around the world. Based on this report the UN Convention on Biological Diversity (CBD) and other policy makers started advising the components of the economy to emphasise the importance of sustainable biodiversity. The “Climate neutral Europe 2050” is one of them to mention. Biodiversity loss can be transmitted to financial, economic and health loss. Thus, different instruments are in place to assess the corporate risk and also to examine if the corporate is able to sustain itself in the long run by maintaining biodiversity. We find that these policy changes before the pandemic might have influenced many companies to report about their non‐financial activities more than before in their business report to maintain their reputation in the financial market. Nature‐friendly business reporting can create brand loyalty for the company and their sustainable trade can reduce the loss of biodiversity, which is beneficial for society. In addition, we propose that if the companies report in detail about their biodiversity related activities, that might influence their peers and competitors in the market to do better for the biodiversity, that might reduce the chance of severe pandemic in future and can attract investors for the companies, which will allow them to invest in highly expensive biodiversity related activities.

The findings of the study will contribute to academic literature related to biodiversity, business reporting and sustainable business model. Additionally, the findings will attract companies to apply a sustainable biodiverse business model, which will enhance brand loyalty, which in turn may attract high investment for the growth prospect of the company. The policymakers will be able to achieve the climate neutral targets. Companies can contribute to the economy by providing transparent information about their awareness of biodiversity in their business reports. Additionally, we contribute to the extant literature by presenting the application of the actor network theory (ANT) during the Covid‐19 crisis. This is because the application of ANT can allow the business to produce a comprehensive and structured annual report, which can be widely used by business stakeholders. This research note provides a contribution in focusing on a direct channel in which integrated reporting is important for economic outcomes, especially during crises. This suggests that business suffering from the Covid‐19 crisis must recognise nature and ecosystems (Hassan, Nandy, & Roberts, 2020). Businesses should also inform stakeholders how they protect the environment, including biodiversity and extinction of species to continue their activities with the stakeholders. We also aim to make practical contributions to the field of sustainability accounting by deriving implications of critical business outlook about the ecosystems.

In the literature we find that after any “Black Swan Event”, there is a tendency among people to change their thought process, where they start believing that if more could be known about the event then the adverse impact of such events can be minimised (He & Harris, 2020). Following this literature, we expect that there will be an urgent call for a better understanding of the underlying causes of Covid‐19 and how various stakeholders of the society could play an important role to avoid such incidents in future. Thus, we focus on one of the valuable stakeholders of the economic system; the companies and propose how these companies can express their accountability towards the environment and society and can assist the society to gain confidence in the post covid‐19 period.

The method employed by our research note is a review of the current research concerning non‐financial business reporting practices, Covid‐19 related publications that linked the role of humans and businesses to biodiversity loss and the development of Covid‐19 and IR with a focus on ecosystems and biodiversity reporting in association with sustainable development. Through this method, we suggest a framework that show the interplay between financial and non‐financial reporting to mitigate future zoonotic diseases such as Covid‐19. We believe that our research note is responding to the most recent call to action that global governments have agreed a vision of “Living in harmony with nature” by 2050. This is one of the United Nations Sustainable Development Goals targets of the 2030 Agenda. We also believe that this integrated thinking of both financial and non‐financial reporting will contribute to the way forward for sustainable development for society. It is very important to mention that the findings of this research notes explained presented throughout this research note. This includes the importance of human reporting, the theoretical framework suggested and the way forward. The study suffers from the limitation of data. So, we focus on published literature to understand how the reporting of non‐financial items in the business report helped the companies to survive during the pandemic and what lesson the peers can learn.

The rest of this research note is organised as follows: In the second and third section, we discuss the literature review and theoretical framework, respectively. In the following sections, we highlight the importance of non‐human reporting by companies and also mention the way forward for company reporting. We summarise out thoughts in the conclusion section.

## 2 LITERATURE REVIEW

According to The Global Reporting Initiative (GRI, 2007), every company makes use of biodiversity resources in their business. Thus, the various stakeholders of the company (society, policy makers, investors, customers, environment etc.) are affected by biodiversity (Lucas et al., 2020) and expect a transparent report of all direct and indirect activities of the company associated with biodiversity in their annual business report. When the companies fail to report clearly about their responsibility towards society, or there is no evidence of CSR in the report, the stakeholders make companies accountable (Jones & Solomon, 2013). To consider a company's accountability towards loss of biodiversity was first proposed in the UN Convention on Biological Diversity (hereafter CBD) in 1992. To expedite the process of considering stakeholders in business models, the UN named the current decade (2011–2020) as the “Decade on Biodiversity” (CBD, 2010). Following the same principles, the European Union (hereafter EU) proposed various targets for companies to achieve during different time periods. During the Covid‐19 outbreak, the EU proposed to take a legislative step to introduce a new requirement of a company's business reporting (European Commission, 2020). Reuters mentioned that there is a growing need for a comprehensive non‐financial report from companies during the Covid‐19 recovery period which will support the companies to achieve their financial targets and long‐term growth prospects. The above statement raises the question that why there is an urgent need for reporting of non‐financial activities of companies during Covid‐19?

Various countries started an environmental management system (hereafter EMS), through which the companies remain responsible towards society and use the biodiversity activities in their reporting. For example, the Companies House introduced the ISO14001 certified EMS in 2002 to monitor the environmental performance of companies. But as socially responsible activities are voluntary, thus, the reporting of social responsibility is not detailed for many companies. The need for reporting on non‐financial aspects has been highlighted by various institutions like GRI, the United Nations Global Compact (hereafter UNGC) etc. along with academic researchers (Haque & Ntim, 2018). Both in academic literature and practice, we find that if a company is not mentioning their environmental, social, and governance (ESG) activities then they lose advantages associated with competition in the financial market and expose them to financial risk (OECD, 2019). Thus, from the above discussion, it is clear that the companies were aware of the importance of the non‐financial activities and its report but still, it is not clear why the policy makers are proposing more importance on non‐financial report, especially during the Covid‐19 recovery period?

The cause of the Covid‐19 crisis is considered very different from the previous great depression or the financial crisis of 2008 (Bernanke, 2020; Reinhart, 2020). IPBES (2019) mentioned that any loss of biodiversity will disturb the ecosystems which will then create a greater risk to all components of the economy. Deforestation, climate change, extinction of species etc. create imbalances in our ecosystem, which allow the spread of pathogens and generate a higher possibility of transmission of diseases to human being and livestock (Hassan, Nandy, & Roberts, 2020). Thus, the EU report (2019) states “It is important – now more than ever ‐ to address the multiple and often interacting threats to ecosystems and wildlife to buffer against the risk of future pandemics”. Paul Polman (Moore, 2020) argues that companies should focus on activities that will not destroy natural capital to invite more pandemic like Covid‐19. To attract bankers for high growth project (Nandy & Lodh, 2012) and to maintain their reputation, companies are encouraged to provide more non‐financial information in their annual report to present their socially responsible image.

In the next section, we will explain how the theoretical models can be related to the importance of reporting of non‐financial activities in annual report and support that such practical changes in business reporting can mitigate the risk of a future pandemic.

## 3 THEORETICAL MODEL

The development of a well‐functioning market is most essential to companies after the financial crisis (Lins et al., 2017). Evidence suggests that businesses engage in profit‐maximising CSR (e.g., Servaes & Tamayo, 2013) to reward for their commitment to society (e.g., Alshbili et al., 2019, 2021; Alshbili & Elamer, 2020; Birindelli et al., 2019; Elamer et al., 2020; Elmagrhi et al., 2019; Ferrero‐Ferrero et al., 2015; Jo & Harjoto, 2011). Others find that CSR is not that important to business during the crisis (Cornett et al., 2016). The literature cannot capture a holistic view of the usefulness of non‐financial reporting. This paper addresses this gap in the extant literature by introducing the Actor Network Theory (hereafter ANT) by identifying the application of the theory during the recovery of the Covid‐19 crisis.

ANT is an analytical lens that aims not to distinct the world into the two domains of society and nature (Barter & Bebbington, 2013; Harman, 2009; Latour, 2004, 2005, 2010; Lee & Stenner, 1999). According to the theory, humans and non‐humans are interlinked (O'Connell et al., 2009; Steen et al., 2006). As a result, ANT brings all entities within its systematic view (humans and non‐humans) and explicitly sets out to “clear the state of nature‐culture dualism” (Ivakhiv, 2002, p. 391). ANT is about giving “due consideration and recognition of [both] the non‐human and human” (O'Connell et al., 2009, p. 20) in the analysis. Indeed, ANT is about showing how humans and non‐humans are interconnected and focuses on relationships between entities (Lee & Hassard, 1999; Lowe, 2001; O'Connell et al., 2009). When using ANT, the analyst has to place themselves in the middle of the action where “connections are continuously being made” (Steen et al., 2006, p. 207) and remade, decentre everything and think relationally rather than in separations (Castree, 2002) or bounded as wholes. In sum, ANT brings forward a world of work, movement and flow where everything is a relational field (Barter & Bebbington, 2013). Accounting scholars are using ANT to highlight the role of calculations and/or accounting systems as actants rather than relying only on analysing interactions between human actors. The ANT allows researchers to explain how a variety of actants are deployed in order to enable a new practice, model or system to be adopted (see, e.g., Briers & Chua, 2001; Caron & Turcotte, 2009; Czarniawska, 2009; Emsley, 2008; Whittle & Mueller, 2008, 2010). The studies suggest innovations are not adopted because they are the “best” but because a variety of actants are enrolled (reports, buildings, accounting systems, calculations) and, in doing so, the network around innovation is strengthened, ensuring the innovation is adopted.

The application of ANT is appropriate to address the research question in this study. By applying the ANT, companies can consider the constant networking relationship between the society and profit aspect in their growth model. The absence of a comprehensive theoretical framework in explaining the networking between the human and non‐human elements of the business can be one of the reasons behind a lack of coherent reporting of non‐human elements by companies. A lack of theoretical underpinning sometimes creates a lack of interest among companies to practice any norm widely in the market (Harman, 2009). Thus, we can observe a lack of socially responsible activities by companies. By applying the ANT, a company can establish an important link between the human and non‐human elements in the report which is an urgent need in the post ‐covid‐19 period. The application of ANT in developing the business report in the post Covid‐19 period can explain why the companies will need to identify the link between human and non‐human elements of business. The pandemic helped the business to understand the need for synchronisation of stakeholders with business instead of focusing on profit factors to survive in a difficult time. Thus, one important question raised by business, policymakers and academics is to identify how to invest in socially responsible activities even when they are expensive to achieve long term economic goals. The pandemic proves that there is inequality in various components of the economy in both developed and developing country (Booth, 2020). Such inequality created the need for investment in non‐human element at various level. Activities like lip services or greenwash etc. cannot be enough to avoid any scrutiny by accountants and the public. Thus, the application of ANT to frame a model considering the relationship between human and non‐human element will be suitable for the post covid‐19 period for any country around the world.

In the literature, researchers mentioned the significance of multiple theories in explaining the company's disclosure of the environment and biodiversity (Haque & Jones, 2020). Following this argument, we apply the Natural Inventory Model (hereafter NIM) along with the ANT to explain the research question. The NIM is widely used in the biodiversity accounting literature (Boiral, 2016; Gaia & John Jones, 2017; Jones, 2014). If a company is not reporting about their responsibility towards the society, it creates pressure from stakeholders, which in turn affect the long‐term performance of the company. Thus, by applying NIM in the post Covid‐19, a company can avoid negative thoughts by stakeholders by reporting their biodiversity or social responsibility related activities which will generate higher profit for the company to invest in highly expensive CSR activities (Samkin et al., 2014).

In summary, we propose that the application of ANT and NIM allow companies to consider non‐human with the human element in their business model and can generate higher motivation for the company to report their biodiversity activities in their business report. This comprehensive model proposes that a company's accountability towards society and mentioning the same in a business report will enhance the benefit towards society.

## 4 IMPORTANCE OF NON‐HUMAN REPORTING

Non ‐financial activities are highly linked with human behaviour (Renneboog & Zhao, 2014). Covid‐19 is highly related to human behaviour not because of its relationship with human health but lack of awareness among human stakeholders of a company who are not aware of the non‐human activities of the business. These non‐human and non‐financial activities affect the financial situation in the market. For example, Schwab (2020) in The Daily Mail article on 20 March 2020, reports about the chairman of the Senate Intelligence Committee (Sen. Richard Burr [R‐N.C.]) and three other senators, selling stocks before the Covid‐19 became a global pandemic. Trading on this insider information raises serious concern about the future of the reporting by the business. The Security Exchange Commission (SEC), European Securities and Markets Authority (ESMA), Canadian Securities Administrators (CSA) and other exchange commissions announced an extension of the regulatory filings because of the unprecedented pressure on business created by coronavirus. Reducing executive pay, dividend, and capital expenditure may allow the business to survive from the hardship they are observing from the Covid‐19 but simultaneously, a business should seriously pay attention to the non‐human items like biodiversity, which can mitigate future risk of financial and economic crisis.

Accounting is a mechanism and tool for change and accountants can play an integral part in this effort to make corporate and investors responsible participants in valuing nature and preventing extinction. Accounting has evolved over the years from producing financial statements to communicate with broader stakeholders by the incorporation of environmental information. The International Federation of Accountants (IFAC) say accountants are obligated to act in the public interest (IFAC, 2018). To meet stakeholders' expectations, companies should report on biodiversity and species extinction accountability. Companies must now urgently act to prevent further loss of biodiversity and extinction before it is too late (Atkins & Atkins, 2018). The planet's future is in our hands, yet our hands have not helped (Hassan, Nandy, Roberts, & Elamer, 2020). It is now accepted by society that care must be taken of the environment, knowledge has improved, and it is no longer only eco‐freaks paying attention (Jones, 2014). We are all stewards of the planet and accountants must take the lead in saving the planet immediately (King & Atkins, 2016). Therefore, by paying attention to biodiversity and ecosystem health, companies can recognise the risks and opportunities, anticipate new markets, mitigate their impacts.

## 5 WHAT SHOULD BE DONE IN THE POST COVID‐19 CRISIS? THE WAY FORWARD

The scarcity of resources and their efficient usage are major challenges for the 21st Century (RLI, 2015). Part of this challenge is derived from the linear economy that consists of extracting resources, making a product, using the product, and disposing of waste (Andrews, 2015). However, viewing waste as the end‐state of consumption has contributed to resource depletion and environmental pollution (Geissdoerfer et al., 2017). This also has negative effects on biodiversity loss and species extinction (Atkins & Maroun, 2018; Hassan, Roberts, et al., 2020; Jones & Solomon, 2013) and has led to the spread of pathogens. Thus, the use of the linear economy approach by a company is “increasingly unsustainable” (Andrews, 2015) and there is a need for a circular economy (henceforth CE). The CE‐approach moves away from waste as the end‐state of consumption and focuses on business‐practices where waste is recovered and products are reused (Gregson et al., 2015). CE can create value in two ways: material origin before product‐use or individuals' business. For example, Philips Lighting (2015, 2017) rents the use of lighting out to Dutch airport Schiphol. Paying peruse of products instead of buying them decreases the total consumption of products (Plepys et al., 2015) and driving customers to use products more efficiently (Hafenbrack et al., 2014). This might have very positive effects in terms of eliminating the immense pressure that plastics are having on the oceans, along with the disappearance of rainforests, and land clearing for agricultural purposes. Businesses are also aware of the CE‐transition and the importance of it in serving the long‐term needs of their stakeholders (Ellen MacArthur Foundation, 2013). The recent study of Gusc, (2019) explored how accounting could be changed to accommodate the transition towards a CE, focusing on how reporting could be improved to better fit the CE, how CE‐value can be measured and how a change in accounting for the CE could take place.

Proponents of the circular economy call for a transformation of current corporate practices from a linear “take‐make‐waste” into an infinite loop where waste and pollution are “designed out,” materials are continually (re)used and natural systems are protected (Ellen Macarthur Foundation, 2019). Academically, it can be defined as “an economic model wherein planning, resourcing, procurement, production and reprocessing are designed and managed, as both process and output, to maximise ecosystem functioning and human well‐being” (Murray et al., 2017, p. 377).

We do believe that with the recent Covid‐19 crisis, businesses should think strategically to implement/adopt the circular economy concept and the CE‐transition should not be hindered by obstructions in the current business environment (RLI, 2015). We also believe that, since accounting serves the important economic function of measuring and communicating an organisation's performance (Bushman & Smith, 2001), it should contribute to society by adopting CE. As such, rethinking accounting frameworks and guidelines is needed (SER, 2016) and accounting professionals need to know how they can incorporate the CE into their activities.

**FIGURE 2:**
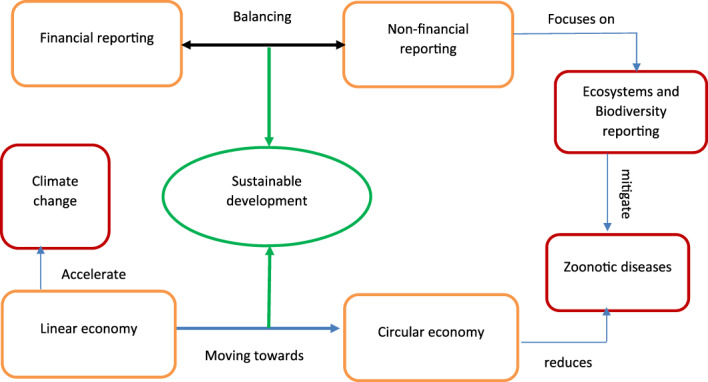
*Source:* Authors [Colour figure can be viewed at wileyonlinelibrary.com]

It is very important also to mention that IIRC introduced the concept of “Value Creation” to prepare integrated reports. The IIRC introduced the value creation model as a system of transforming inputs through its business activities into outputs and outcomes, which aim to fulfil the organisations' strategic purposes and create value over the short, medium and long‐term (IIRC, 2013). We do believe that this change is so important than ever to consider two main important issues. (1) to implement/adapt the CE concept and (2) to report on biodiversity and extinction accounting in a more structured and mandatory way via producing integrated reports (IR) that cover both financial and non‐financial /non‐human information to create value on short, medium and long terms. This concept can be expanded to include individuals where householders, as part of communities, should implement this concept by replacing recycling with upcycling, in which the latter means creative use of an old item. Figure 2 depicts the interplay between financial and non‐financial reporting to mitigate future zoonotic diseases such as Covid‐19.

Future research should explore how accounting can affect the CE‐transition and how accounting barriers for the CE can be mitigated. The contribution to accounting change literature lies in identifying how accounting professionals feel accounting change for the CE can be accomplished, namely through information‐need and formal‐rule‐setting. The recent news about the determination of the business to run their activities and to support their employees during a difficult time provides positivity and shows the actual duties that businesses have towards society. For example, on 13 March 2020, BT Group Plc says that even if the CEO of the company tested positive for Covid‐19 still it will carry on with their business. Also, the support provided by business as a thank you message to the UK National Health Service (NHS) is an example of supporting the community (Dominos offering free Pizza to NHS workers). Car manufacturers across the world have started converting their plants to produce ventilators and face masks after governments called for help in fighting the coronavirus pandemic (Lovett, 2020). Many brand businesses like Prada, Gucci, Yves Saint Laurent and Balenciaga, Zara among others are shifting their focus by postponing the production of their own products to the most important items during this crisis which are face mask and hand sanitiser (Bramley, 2020). The immense support from the business community gives us hope that for the welfare of human beings the business community will pay attention to non‐human activities along with their financial activities and will try to provide more information about both in future to generate more awareness in the society. An initiative by business to balance between financial and non‐human items in their reporting in future is the most ideal situation we can expect for. We propose revised legislation from every government to take care of non‐financial activities done by business for the safety of human health and to mitigate the risk of a future pandemic.

## 6 CONCLUSION

In this paper, we critically reviewed the learning about the most devastating crisis generated by Covid‐19. The method adopted by our research note is the review of the extant literature on non‐financial business reporting practices, IR and CE to gain insight into drawing a theoretical framework regarding the balance between financial and non‐financial reporting to achieve sustainable development by moving towards CE and improving ecosystems and biodiversity reporting. We find that the policymakers while focusing on financial requirements and the transparency of reporting by the business, should consider non‐financial aspects like elements of CE, to assist companies to sustain for the long term in the economy. The attention towards the importance of non‐human aspects in business reporting is a quite recent event. Thus, we argue that the human and non‐human aspects are considered separately, and the non‐existence of any comprehensive business model is not enough to generate awareness among the stakeholders of the business. We find evidence from the literature that the Covid‐19 crisis could be a result of ignorance of non‐human activities at large (Ceballos et al., 2020). Without proper accountability of the company towards the society and explaining their activities to the stakeholder to attract more investment to their costly CSR activities could generate more pandemics in future.

In this encouraging piece of study, we recommend an urgent need for mandatory integrated reporting by the business. We suggest the combination of ANT and NIM theory may assist companies to better explain how to include a non‐human element in their business reporting. In addition, we propose that the adoption and implementation of the circular economy can enhance stakeholders' credibility and trust and that can create value in the short, medium and long term for the company in the post Covid‐19 period. The transformation for the business to implement CE can become a game‐changer by significantly reducing pressure on the planet, biodiversity loss, extinction and support a sustainable future for all.

The recommendations in this research will provide guidelines to regulators about the importance of creating awareness of biodiversity and extinction accounting among the business community. We suggest that well‐designed capital distribution between financial and non‐human activities can reduce the probability of systematic risk in the economy. This finding is in line with the study by Gauthier et al. (2012), where they prove that the market risk can be reduced by 25% when there exists proper planning about the capital requirements. Also, our results are important for the accounting regulators to enforce mandatory integrated reporting, which can create a circular economy in practice.

Our research note provides opportunities for future research. For example, future research should explore how accounting can affect the CE‐transition and how accounting barriers for the CE can be mitigated. The contribution to accounting change literature lies in identifying how accounting professionals feel accounting change for the CE can be accomplished, namely through information‐need and formal‐rule‐setting. In addition, the immense support from the business community gives us hope that for the welfare of human beings, the business community will pay more attention to non‐financial activities along with their financial activities in future to generate more awareness in society. We propose revised legislation from every government to take care of non‐financial activities done by business for the safety of human health and mitigate the risk of future pandemics.

Our research note provides practical implications for policy makers to develop guidelines to regulators about the importance of creating awareness of biodiversity and extinction accounting among business community. Our results are important to accounting regulators also to enforce integrated reporting mandatory rules and regulations and to issue some guidelines on how to implement a circular economy. The contribution to accounting change literature lies in identifying how accounting professionals feel accounting change for the CE can be accomplished, namely through information‐need and formal‐rule‐setting.

Due to the recent Covid‐19, there is no data available to analyse. As other research, this research also has limitations. There are huge opportunities for future research to tackle this crisis. For example, a stream of research might focus on initial initiatives for adopting a circular economy in different sectors. Another stream might concentrate on how the mandatory adoption of integrated reporting will enhance stakeholders' trust and credibility. Using the existing available data and by studying the changes in firms' reporting, we need to conduct additional research to identify the socially optimal model of capital allocation by a business. More attention is required to understand how awareness among business about biodiversity can be enhanced by reducing the complexity of the application of the policies and how the policymakers can affect the culture associated with business reporting.

## Endnotes

^1^
See World Health Organization (WHO) website for details https://www.who.int/health-topics/coronavirus#tab=tab_1.

^2^
See Figure 1.
